# Temporal Encoding to Reject Background Signals in a Low Complexity, Photon Counting Communication Link

**DOI:** 10.3390/ma11091671

**Published:** 2018-09-09

**Authors:** Alexander D. Griffiths, Johannes Herrnsdorf, Christopher Lowe, Malcolm Macdonald, Robert Henderson, Michael J. Strain, Martin D. Dawson

**Affiliations:** 1 Institute of Photonics, University of Strathclyde, Glasgow G1 1RD, UK; johannes.herrnsdorf@strath.ac.uk (J.H.); michael.strain@strath.ac.uk (M.J.S.); m.dawson@strath.ac.uk (M.D.D.); 2 Department of Mechanical & Aerospace Engineering, University of Strathclyde, Glasgow G1 1XJ, UK; christopher.lowe@strath.ac.uk (C.L.); malcolm.macdonald.102@strath.ac.uk (M.M.); 3 CMOS Sensors & Systems Group, University of Edinburgh, Edinburgh EH9 3JL, UK; robert.henderson@ed.ac.uk

**Keywords:** LED, GaN, single-photon avalanche diode, optical communications, CubeSats

## Abstract

Communicating information at the few photon level typically requires some complexity in the transmitter or receiver in order to operate in the presence of noise. This in turn incurs expense in the necessary spatial volume and power consumption of the system. In this work, we present a self-synchronised free-space optical communications system based on simple, compact and low power consumption semiconductor devices. A temporal encoding method, implemented using a gallium nitride micro-LED source and a silicon single photon avalanche photo-detector (SPAD), demonstrates data transmission at rates up to 100 kb/s for 8.25 pW received power, corresponding to 27 photons per bit. Furthermore, the signals can be decoded in the presence of both constant and modulated background noise at levels significantly exceeding the signal power. The system’s low power consumption and modest electronics requirements are demonstrated by employing it as a communications channel between two nano-satellite simulator systems.

## 1. Introduction

Conventional optical wireless communications (OWC) involves the modulation of the optical emission from a light source, such as a light-emitting diode (LED) or laser, and detection of the output light with a photoreceiver [1]. When transmitting over long distances, or through high loss media, received power will become greatly reduced, and eventually be lost in noise from background light or within the receiver electronics themselves. Single photon detection and counting methods are used to achieve high receiver sensitivity with intensity modulated optical signals [2,3,4,5,6]. With the use of forward error correction (FEC) codes and high order pulse position modulation (PPM) [7], photon counting systems can operate with extremely low numbers of photons per bit [8]. In combination with arrayed receivers, the high sensitivity of single photon counting techniques has potential for deep-space communication links, operating at megabit rates [9,10].

The link performance of a single photon counting link can suffer significantly under the presence of noise counts [11], which can occur due to background light in the channel, or dark counts occurring within the detector. These additional counts cause erroneous detection of bits, necessitating the use of powerful FEC codes [2,12]. Photon counting links using coincident photon pairs can overcome noise limitations [13,14,15,16], and can be used for quantum key distribution [17,18,19]. However, such systems typically require high efficiency photon pair sources, putting large form factor requirements on the internal and transceiver optics. In fact, many single photon communication links make use of complex, large, and/or costly equipment, such as cryogenic receivers [3,10], CW lasers with external modulators [2,6] and arbitrary waveform generators [4]. This makes such systems difficult to deploy in application areas where size, weight and power budgets may be limited.

**Figure 1:**
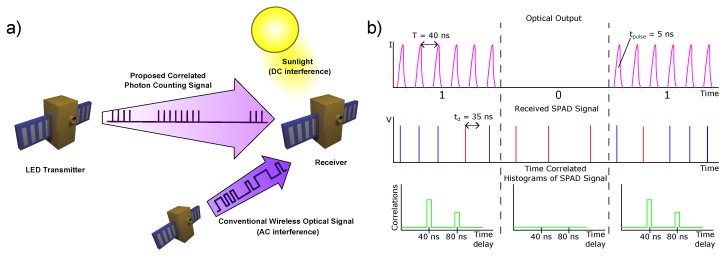
(**a**) Application scenario for inter-satellite communications: the proposed signal encodes data in trains of time-correlated optical pulses and is compatible with low power consumption LED emitters. It is resilient against constant background such as sunlight and also insensitive to most AC background such as conventional wireless optical signals. (**b**) Schematic of the transmission scheme used. LED output on transmission of “0” and “1” (**top**), SPAD response to the LED signal (**middle**) and the calculated correlation histograms for each data interval (**bottom**).

Here, we demonstrate a novel optical transmission scheme, suitable for OWC with single photon detection, requiring a single, low photon flux channel. Compared with existing methods, this scheme is implemented with simple and widely available semiconductor components and electronics. A gallium nitride (GaN) micro-LED transmitter, silicon single-photon avalanche diode (SPAD) receiver and field-programmable gate array (FPGA) electronics provide a compact system and with low power consumption. The transmission method operates under the presence of both constant and modulated background noise, which is enabled by the encoding of data in the timing statistics of the received photons. The following sections discuss the details of the transmission scheme, its current implementation, data transmission results and a demonstration of the system’s suitability for inter-satellite communications, such as shown in Figure 1a.

## 2. Results

### 2.1. Time Correlation Encoding Scheme

The transmission scheme presented here, inspired by time-correlated single photon counting (TCSPC) techniques often used for fluorescence lifetime imaging [20], involves the use of a single SPAD to receive time correlated signals at a single photon level. Analysis of the SPAD response to incoming light over an interval $\left[-t_{1},t_{1}\right]$ shows that the correlation count density function $g\left(\tau \right)dt^{\prime }$ of recording two subsequent SPAD counts with temporal separation in the interval $\left[\tau ,\tau +dt^{\prime }\right]$ is given by:

(1) $$g\left(\tau \right)=\int_{-t_{1}}^{t_{1}}dt\;f\left(t\right)f\left(t+\tau \right).$$

Here, f(t) is the temporal probability distribution of received SPAD pulses, which is determined by the optical signal from the transmitter. Full analysis is given in the Supplementary Materials of Reference [21]. If a suitable optical source transmits pulses with a time separation of *T*, g(τ) will show a peak at τ=T, as the probability of observing SPAD pulses separated by *T* is increased. Equation (1) is the autocorrelation of f(t), so it is expected that peaks in g(τ) would have a width of $2t_{pulse}$, where $t_{pulse}$ is the width of the optical pulse. After detection of a photon, the output of a SPAD has a “dead time” ($\tau_{d}$) in which it is insensitive to further photons, typically 10 s of ns in length [6,12]. It is important that $T>\tau_{d}$, as otherwise the SPAD would not recover from the first pulse in time to see the second. This restriction can be lifted by using a SPAD array [22,23], however here we consider the use of only a single SPAD. The presence and/or temporal position of peaks in g(τ) directly depends on the sequence of optical pulses from the transmitter, and therefore can be used as a means of transmitting data.

In reality, the SPAD output is not a continuous probability distribution, but a series of discrete photon detection events. These events can occur due to the optical pulses from the transmitter, background photons, or dark counts. SPADs typically have dark count rates (DCRs) ranging from 100 s of Hz to several kHz [2,6,7,12], however, the SPAD used in the following experimental sections has an active cooling system, reducing the DCR to 25 Hz. The SPAD output signal will be sampled over a time period, into $N_{s}$ time bins $t_{i}$, $i=1,\ldots ,N_{s}$, chosen to be smaller than $\tau_{d}$, so that bin contains a number of counts $f_{i}\in \left[0,1\right]$. The correlation time will also be discretised into $\tau_{j}$, $j=1,\ldots ,N_{\tau }$. For a single pair of pulses a correlation either is or is not detected, so the optical signal must be repeated many times to distinguish correlation counts from noise. Instead of a single pulse pair, the pulses are continuously repeated at a rate specified by the temporal separation $R_{pulse}=1/T$. With correlation time bin size chosen as an integer multiple of sampling bin size, $\tau_{bin}=kt_{bin}$, we can define start and stop indices for correlating across *i* as $n_{start}=\tau_{1}/t_{bin}$ and $n_{stop}=n_{start}+kN_{\tau }-1$. With this, the discrete form of Equation (1) is:

(2) $$g\left(\tau_{j}\right)=\sum_{i=1}^{N_{s}-n_{stop}}\sum_{l=0}^{k-1}f_{i}f_{i+n_{start}+\left(j-1\right)k+l}.$$

As $f_{i}$ is a binary value, and the output from the SPAD is a transistor-transistor logic (TTL) signal, the summation could be implemented with simple logic circuits.

Encoding data in $g(\tau_{j})$ has the potential to allow data transfer at exceptionally low light levels, and in the presence of significant background illumination. To detect correlations, the receiver requires the detection of a single photon from each optical pulse. Such conditions allow average received power to be extremely low, in the range of pW. The trade-off in this transmission scheme is that the data rate is expected to be relatively modest, as the optical signal must be repeated several times in order to generate a distinguishable signal in $g(\tau_{j})$.

There are several potential ways to encode data in $g(\tau_{j})$, with parallels to on-off keying (OOK), PPM or pulse amplitude modulation (PAM). Here, we consider the simplest form of encoding, OOK, where data can be encoded using a single pulse time separation. On transmission of the symbol “1”, pulses are transmitted continuously with a fixed time separation T=40 ns, corresponding to a repetition rate of 25 MHz, so $g(\tau_{j})$ will show a peak at τ=T. This time separation has been chosen as the dead time of the SPAD used is 35 ns. On transmission of the symbol “0” no pulses are transmitted, producing only background counts in $g(\tau_{j})$. A schematic of the expected waveforms is shown in Figure 1b. With pulse width $t_{pulse}=5$ ns, deliberately less than $\tau_{d}$, only one signal photon can be detected from each pulse, indicated by the blue SPAD signals in Figure 1b. In reality, the detection rate will be less than one per pulse, and pulses can also be missed if they are received during the dead time after a noise pulse, indicated in red. Time correlation of the measured events from the SPAD is performed over a data interval, producing a histogram with peaks at 40 ns intervals for transmission of a “1”, and a background correlation level for transmission of a “0”, determined by ambient background light and detector dark count rate. Applying a threshold to the histogram bin generated for each symbol at a delay of 40 ns allows decoding of the binary stream. This threshold will have to be sufficient to reject correlation counts from background and dark count correlations. A crucial feature of this method is that it is robust to temporal jitter between the transmitter and receiver. Synchronisation of the system can be easily achieved by using an embedded clock in the transmitted data, as discussed in Section 4.2.

### 2.2. Experimental Demonstration

**Figure 2:**
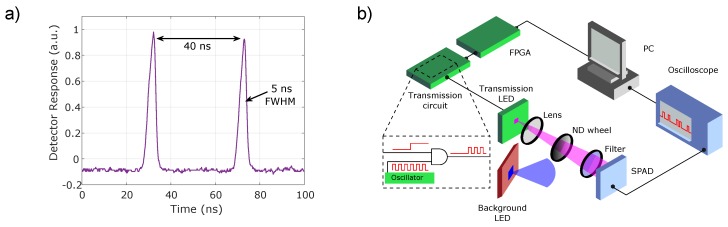
(**a**) Measured pulse pair from a micro-LED source. (**b**) Schematic of the experimental setup.

The scheme detailed above was realised using a GaN violet emitting (405 nm), micro-LED device as the transmitter and a silicon Single Photon Avalanche Detector (SPAD) as the receiver. The LED chip was bonded to a custom CMOS driver allowing short pulse operation, with durations of 5 ns. The LED wavelength of 405 nm was initially used as the devices showed improved pulsed performance in this configuration over other wavelength counterparts. Data signal modulation was applied as a slower on-off keying of the short pulse train. Figure 2a shows a measured pair of pulses from the micro-LED of 5 ns duration and with a relative delay of 40 ns. A variable neutral density filter was placed between the emitter and detector to control the received power at the SPAD. A schematic of the measurement setup is shown in Figure 2b. Full details of the devices and electronic drivers are given in Section 4.1.

### 2.3. Signal-to-Noise Ratio

To set an operation threshold for the system, a figure of merit similar to the classical signal-to-noise ratio (SNR) must be defined. In this method, it is the distinguishability of the correlation peak in the g(τ) function that indicates the robustness of the classical information recovery to noise. Conventional SNR can be defined as $SNR=10\log_{10}\frac{N_{signal}}{N_{noise}}$, where $N_{signal}$ is average signal correlation counts and $N_{noise}$ is average noise correlation counts. However, as the number of pulse repetitions increases, the correlation counting interval increases, causing both $N_{signal}$ and $N_{noise}$ to increase at linear rates. This results in a constant SNR, which does not reflect the observed increase in distinguishability of signal correlations with increasing pulse repetitions.

**Figure 3:**
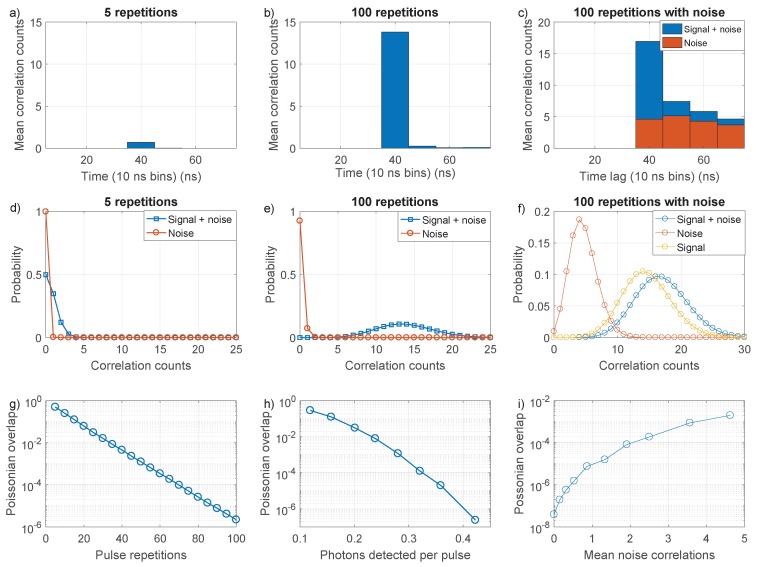
Measured correlation histograms for: (**a**) 5 pulse repetitions; (**b**) 100 pulse repetitions; and (**c**) 100 pulse repetitions with background noise. Poissonian distributions for the signal and noise correlation counts for: (**d**) 5 pulse repetitions; (**e**) 100 pulse repetitions; and (**f**) 100 pulse repetitions with background noise. (**g**–**i**) Poissonian overlap according to Equation (3) as a function of: (**g**) number of pulse repetitions; (**h**) average number of photons detected per pulse; and (**i**) average number of background noise correlations.

Instead, it is more useful to consider the statistical distribution of correlation counts for signal and noise. Photon counting experiments were undertaken using the experimental setup described above, with a received power at the detector of 38 pW, corresponding to a detector count rate of $1.07\times 10^{7}$ Hz, in a dark lab environment with an average background count rate of 619 Hz. Note that this count rate contains both dark counts and counts from the small amount of ambient light. The delay correlations of detected photons were binned with a resolution of 10 ns, with the transmitted pulse delay set at 40 ns. Figure 3a,b shows average histograms of received photon correlations for 5 and 100 pulse repetitions, respectively. Figure 3c shows the histogram for 100 pulse repetitions under high background conditions, displaying the correlation histogram due to background noise alone, and signal with noise. The background count rate for this measurement was $10^{7}$ Hz.

In Figure 3a–c, the signal is defined as the number of correlations in the 40 ns delay time bin, and the noise correlation count is taken from the 60 ns delay bin. Correlation counts follow a Poissonian distribution, as they are discrete independent events arising directly from shot noise limited photon counts. Figure 3d,e shows the measured Poissonian distributions for signal with noise and noise alone correlation counts at 5 and 100 repetitions of the pulses, respectively, taken from 1500 independent measurements of each case. Figure 3f shows the distributions for background noise, signal alone (identical to Figure 3e) and combined signal and noise.

At five repetitions, the probability distributions for signal and noise are strongly overlapped. Thus, a correlation count peak due to signal transmission is difficult to distinguish from a correlation count peak due to random background and dark counts. At 100 repetitions, the overlap of signal and noise distributions is significantly reduced, making distinction much easier. A histogram threshold equates to a point along the x-axis of the distribution plots. Evidently, a threshold of 2 would result in many erroneous detections at five repetitions, whereas, at 100 repetitions, the majority of signal correlation peaks would be correctly identified, and noise correlation peaks rejected. Under high background noise conditions, the number of correlations from noise is increased, so the threshold must increase to distinguish between the correlations due to noise from those due to the signal, as both will be present on transmission of “1”.

Distinguishability is therefore described by Equation (3), the overlap of the Poisson distributions for: (i) the total signal and noise contributions, $P_{T}\left(k\right)$; and (ii) the noise alone, $P_{n}\left(k\right)$. $P_{T}\left(k\right)$ is related to the signal count distribution, $P_{s}\left(k\right)$ and noise count distribution, $P_{n}\left(k\right)$ via Equation (4). Here, $P_{n/s}\left(k\right)$ are the the probabilities of *k* correlation counts occurring due to noise or signal with mean λ, given by Equation (5).

(3) $$\begin{array}{c} Overlap=\sum_{k=0}^{\infty }P_{T}\left(k\right)P_{n}\left(k\right), \end{array}$$

(4) $$\begin{array}{c} P_{T}\left(k\right)=\sum_{m=0}^{k}P_{n}\left(m\right)P_{s}\left(k-m\right). \end{array}$$

(5) $$\begin{array}{c} P_{n/s}\left(k\right)=\frac{\lambda^{k}e^{-\lambda }}{k!} \end{array}$$

Figure 3g–i shows the calculated overlap for changing pulse repetitions, photon detection rate and noise correlations, respectively. The overlap reduces exponentially with pulse repetitions, faster than exponential with photon detection rates, and increases sub-exponentially with background correlations. This is understood by noting that λ in Equation (5) follows $\lambda =p_{ph}^{2}N_{rep}$, where $p_{ph}$ is the probability of detecting a photon from a single pulse, and $N_{rep}$ is the number of pulse repetitions.

Therefore, the distinguishability of binary 0 and 1 is governed by the Poissonian overlap, Equation (3), and depends on the number of sampled pulse repetitions, the received signal power, and the background intensity, with the first two parameters dominating.

### 2.4. Data Rates

**Figure 4:**
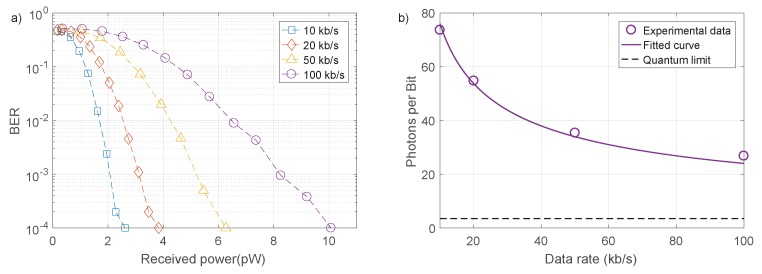
(**a**) BER as a function of received signal power for varying data rates. (**b**) Received photons per bit required to achieve a BER of less than $10^{-3}$ for varying data rates, fitted with a $x^{-\frac{1}{2}}$ relationship. The standard quantum limit for OOK at this BER is also shown.

**Figure 5:**
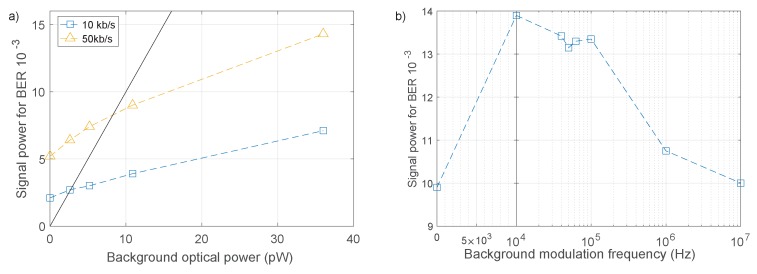
(**a**) Required signal power to attain a BER of $10^{-3}$ under constant background power for 50 and 10 kb/s. Equal signal and background power is indicated by the solid line. (**b**) Required signal power to attain a BER of $10^{-3}$ under modulated background conditions. Required power increases by approximately 40% when background modulation rates are comparable to the signal data rate.

The achievable data rate of this system is determined by the number of pulse repetitions required to distinguish the signal, and hence the received power and the time separation between pulses. The SPAD response imposes a lower limit on this separation, due to the dead time $\tau_{d}$ and pulse width, $\tau_{pulse}$, giving an achievable data rate of:

(6) $$R_{data}=\frac{1}{N_{rep}\left(t_{pulse}+\tau_{d}\right)}.$$

where $N_{rep}$ is the number of pulse repetitions required to see a distinguishable peak in the correlation histogram. Use of a SPAD array could lift the restrictions imposed by dead time through pulse combining techniques [23]. To demonstrate the system performance as a function of received power and data rate, bit error ratio (BER) measurements were taken. A target BER of $1\times 10^{-3}$ was used, as FEC codes can be used to reduce this to effectively error free levels at a small overhead on data throughput [24]. A pseudo-random bit sequence (PRBS) of $10^{4}$ bits was transmitted, limited by the data processing capabilities of the oscilloscope and PC components in the measurement setup. The ND filter wheel allowed control of received power, or equivalently, photon detection probability. Figure 4a shows BER curves for varying data transmission rates, taken with minimal background light. At 8.25 pW of average power, corresponding to an average of 0.34 incident photons per pulse, a data rate of 100 kb/s was possible with a BER of less than $10^{-3}$. Received optical power can be reduced at the expense of data rate. A data rate of 10 kb/s can be achieved at the same BER with 2 pW, corresponding to 0.08 photons per pulse. The power measurements quoted here and used in Figure 4 and Figure 5 are the incident optical power on the active area of the SPAD, calculated through numerical methods from the average detector count rate. Detector count rate is the parameter which governs BER performance, however the incident optical power will be influenced by the performance of the SPAD. Most importantly, the photon detection probability (PDP) at 405 nm is 18%, so the incident photon flux is significantly higher than the detector count rate. More efficient photon detection would improve BER performance in terms of required power.

The system performance can also be described in terms of the number of received photons per bit. Figure 4b shows detected photons per bit for each data rate, at the level required for a BER of less than $10^{-3}$. The fitted curve is calculated from the relationship between correlation counts, received power and data rate. The number of signal correlations depends on the square of received power and is inversely proportional to the data rate $R_{data}$. To reach a given target BER, a certain constant number of signal correlation counts must be reached, meaning ${\left(ph/s\right)}^{2}\propto R_{data}$. As photons per bit is the required photons per second divided by data rate, the data follows a $y=x^{-\frac{1}{2}}$ relationship. The 100 kb/s link is transmitting each bit with an average of 27 detected photons. This is relatively close to the standard quantum limit (SQL), set by Poissonian photon statistics [25]. For a BER of $10^{-3}$, a minimum of seven photons is required to detect a “1”. Therefore, an average of 3.5 photons per bit is required, assuming the probability of transmitting “0” or “1” is equal. The implemented scheme will be unable to reach the SQL, due to the correlation approach. Two photons are required for a single correlation detection, which itself has a Poissonian distribution that must be distinguished from noise.

After correcting for detector efficiency, 27 detected photons equate to $7.37\times 10^{-17}$ J incident on the detector per bit. This exceptionally low energy demonstrates the suitability of the transmission scheme in low power or high loss systems. As mentioned above, more efficient photon detection would allow further reductions in energy received per bit. Additionally, efficiency improvements will be possible through use of PPM style transmission, to transmit multiple bits per correlation peak.

### 2.5. Robustness to Noise

A major advantage of this transmission scheme is that it is expected to be robust against background counts, as ambient light is generally uncorrelated on the time scale of 10 s of ns. To verify this, BER measurements were taken for increasing levels of background light using a secondary 450 nm light source, as shown in Figure 2b. As background counts increase, the probability of detecting noise correlations increases. The threshold applied to the correlation histogram must be increased to avoid erroneous detection of bits, requiring higher received average power to maintain the same BER performance.

The results in Figure 5a show the incident optical power required to maintain a BER of $1\times 10^{-3}$ for 10 and 50 kb/s with increasing levels of background optical power. The signal power requirements do increase, however, they are still very low. At high background levels, the required signal power is significantly lower than the power received from background illumination, with equal levels indicated by the solid line. Here, the interplay between number of pulse repetitions, photons detected per pulse and background counts becomes important. As discussed in Section 2.3, higher levels of background power increases the number of bit errors, as the overlap of Poisson distributions increases. While the target BER can be recovered by increasing received photons per bit, equivalent to increasing the signal power, it can also be recovered by increasing the number of transmitted pulses, equivalent to reducing the data rate. The result is that given a certain level of background optical power, a signal can always be transmitted at a power level below that of noise, at the expense of data rate.

The system was also measured under modulated background illumination. Since the number of detected correlation counts depends on the square of received power, a high modulation rate background should interfere in the same manner as a DC signal at the root-mean-squared (RMS) of its count rate. For this reason, background signals were set to maintain similar RMS photon count rates for comparison to DC measurements. The RMS background optical power was approximately 15 pW for all measurements. The power required to maintain a BER of $10^{-3}$ is shown in Figure 5b, and displays two distinct groups of results. The high background modulation rates of 1 and 10 MHz show similar required signal power to constant background conditions, while when the background modulation rate is close to the correlation link data rate, the BER performance is degraded, requiring approximately 40% more received power. This reduction in performance occurs as the background signal is now generating different levels of noise correlations from one bit period to the next, making it more difficult to choose a correlation threshold. At higher background modulation rates the background signal completes many cycles within a single bit period of the correlation link, and the dead time of the SPAD restricts the number of photons that can be detected per background cycle, causing the signal to interfere in the same manner as constant background. Nevertheless, all conditions still reach a BER of less than $10^{-3}$ for less than 14 pW of received signal power.

Under all background conditions, the signal is transmitted with a lower photon count rate than the background signal, demonstrating low power performance even with high power modulated background interference.

### 2.6. Satellite Systems Demonstration

The communications system presented here is applicable in many scenarios, but is particularly attractive for inter-satellite links. The semiconductor devices are extremely compact, have low power consumption and are readily integrated with control electronics. LED based visible light communications shows potential for use with cube satellites [26,27]. The robustness of the signal to background noise and operation at picowatt levels of received optical power means the scheme could be implemented without the high accuracy pointing requirements, telescope optics and filters of current satellite systems.

**Figure 6:**
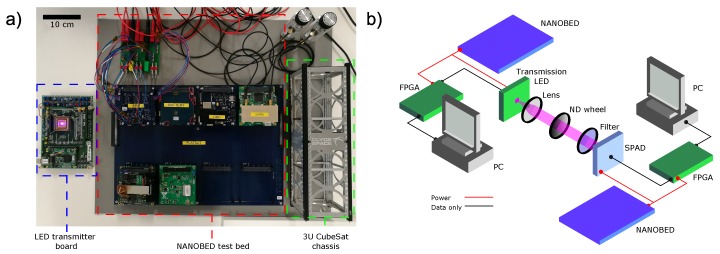
(**a**) Photograph of the micro-LED transmitter board (left), NANOBED “Flatsat” test bed (middle) and a standard 3U CubeSat chassis (right). (**b**) Schematic of the experimental setup for real time data transmission under simulated satellite power.

To highlight this capability, the system was tested in the nano-satellite hardware and software test-bed, NANOBED, shown in Figure 6a, which can simulate the available power systems on a cube satellite. To demonstrate that the full communication link was able to be powered by the NANOBED, a real time decoder, incorporating embedded clock signal recovery, was implemented on a FPGA platform to replace the oscilloscope and PC components in the characterisation setup. Details of this setup, shown in Figure 6b, are given in Section 4.3.

The LED transmission system was integrated with one NANOBED system, while the SPAD receiver system was integrated with a second. In this work, the solar panel emulating power sources are used to supply the transmitter and receiver devices via the electrical power supply (EPS) and battery units, simulating an in-orbit scenario. The transmitter side of the real time link requires a single FPGA board, from which the CMOS micro-LED array is powered and controlled. On the receiver side, the commercial SPAD module requires a 6 V DC supply, and a second FPGA is used to process the received signals. A summary of typical power consumption is shown in Table 1. The SPAD consumes the most power in the system, however the commercial module has not been designed with power conservation in mind, and employs significant levels of active cooling. A custom SPAD receiver may show power requirements of 10–100 mW [6,22]. While the lack of active cooling would result in higher levels of dark counts, the resilience to constant background signals demonstrated in Section 2.5 indicates this would not be problematic. Additionally, bespoke electronics in place of FPGA boards may also permit lower power consumption, therefore this demonstration should be thought of as an upper limit on power requirements.

**Figure 7:**
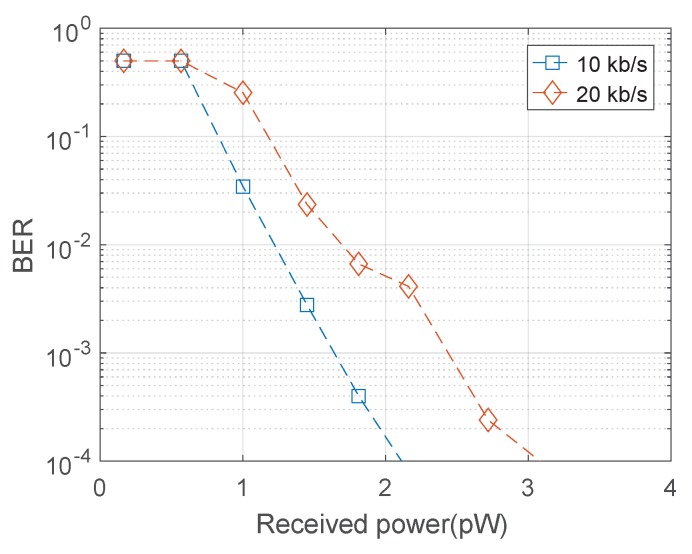
BER performance of the real-time communication link under simulated satellite power.

For the laboratory demonstration, the transmitter and receiver were placed 4 m apart with the micro-LED pixel projecting the light across a 4 cm wide square with received power controlled using a neutral density filter. A micro-LED emitter at 450 nm was used to improve the PDP to 25%. As shown in Figure 7, the live link requires 2.5 pW of received power to maintain a BER of $10^{-3}$ at 20 kb/s. On a 20 μm diameter SPAD, 3 pW corresponds to an intensity of 9.5 mW/m${}^{2}$. To provide this over the projected 4 cm wide square, 15.3 μW must be collected from the micro-LED by the transmitter lens.

## 3. Discussion

We have demonstrated a transmission scheme suitable for single photon level optical wireless communications. By transmitting temporally correlated signals, data communications can be performed at extremely low light levels, with received power on the order of pico-Watts. Signals can be transmitted using an LED, and received with a single SPAD. A 100 kb/s link has been achieved with a BER of less than $10^{-3}$ at a received power of 8.25 pW. The scheme is robust to background light, with only a minor increase in required power for very high background conditions. Modulated background signals appear to have little additional influence over that of continuous background, suggesting the scheme could be used in parallel with other optical communications with minimal interference. Furthermore, multiple transmission systems using this scheme could operate without interfering with each other, simply by using different pulse time separations. The modest data rates presented in this work are dominated by two key factors, firstly the requirement of this protocol for correlating many repetitions of a pulse pattern, and, secondly, the dead time of the SPAD detector itself.

A real time transmission setup has been demonstrated, showing a method for clock synchronisation and determination of a threshold level. The current, unoptimised implementation allows a data rate up to 20 kb/s, with only a minor reduction in performance when compared to offline processed transmission. The real time transmission link has been demonstrated in a simulated satellite environment, providing data transmission at a received optical intensity of 9.5 mW/m${}^{2}$ under simulated nanosatellite power systems. Additionally, GaN LEDs at low current densities show higher wall-plug efficiencies than laser diode counterparts [28], further enhancing the power consumption characteristics of the system. In future satellite-focussed experiments, an optimum transmission wavelength will be chosen, based on LED performance, detector response and solar-blind wavelengths.

Data rate and photons per bit efficiency can both be improved through relatively straightforward modifications to the system. By using a SPAD array as a receiver rather than a single device, the dead time limitation can be overcome and therefore higher data rates achieved. In addition, by implementing a form of pulse position modulation, with powerful FEC codes, the photons per bit transmission efficiency can be improved. Finally, data rates may be enhanced by using a form of pulse amplitude modulation, however the received power requirement would also increase.

This transmission protocol has clear applications in communications systems for long range or high loss environments, but is also equally applicable in microscopy or low light level imaging systems when coupled with a SPAD imaging array, and can be implemented using a wide range of pulsed optical sources dependent on the application.

## 4. Materials and Methods

### 4.1. Optical Transmitter and Receiver Realisation

The transmitter used for the results presented here is a complementary metal oxide semiconductor (CMOS) integrated gallium nitride micro-LED pixel. Details and fabrication of comparable devices can be found in [29]. The micro-LED pixel is a square 100×100 μm in size, and part of a 16×16 array with a 405 nm emission wavelength. The array was fabricated in flip chip format, and bump-bonded onto CMOS control electronics which allow the LEDs to be modulated in a pulsed mode, triggered by the falling edge of an input logic signal. The shortest stable optical pulses $t_{pulse}$ that could be generated with this device and control system were 5 ns. To produce pulses for the OOK transmission, a data signal was produced at the desired data rate by a field programmable gate array (FPGA) (Xilinx Spartan-3, XEM3010, Opal Kelly, Portland, OR, USA). The FPGA clocks were derived from a 48 MHz signal from a USB microcontroller, with parts of the system running at 100 MHz. The data sequence was sent to a simple transmission circuit. Here, the data signal was combined with an oscillator producing a signal of square waves with a period of 40 ns, through an AND gate, as shown in Figure 2b.

The SPAD receiver is a commercial module (SPCM20A, Thorlabs, Newton, NJ, USA), with a detector active area diameter of 20 μm. The dead time of the detector is 35 ns, and the typical dark count rate is 25 Hz. At 405 nm and 450 nm, the PDP is 18% and 25%, respectively. The module outputs 3 V logic signals indicating photon counts. This signal was sent to an oscilloscope, and collected by the PC for offline processing of $g(\tau_{j})$. In a practical system, this processing could be performed by digital logic circuits.

The LED output was collected with a lens (C220TME-A, Thorlabs, Newton, NJ, USA) and transmitted through a graded neutral density (ND) wheel (NDC-50C-4M-A, Thorlabs, Newton, NJ, USA). A 450 nm shortpass filter was used in front of the SPAD to reject additional background light. This filter was removed for the experiments assessing performance under high background conditions. The pixel was imaged onto the SPAD active area. As the pixel image is approximately a 7 mm square, only a small portion of the light was imaged on to the SPAD circular active area of diameter 20 μm. In a practical system, receiver optics could be used to collect more light on to the active area of the SPAD, reducing the loss through the channel. Received optical power was calculated numerically from the average number of photon counts detected. This method accounts for detector dead time, and photon detection probability at the operational wavelength. Details of the calculation can be found in the Supplementary Materials of Reference [21].

To assess the effects of DC background illumination, a commercial 450 nm LED (LD CQ7P, OSRAM, Munich, Germany) was placed within a few centimetres of the transmitter LED, directed towards the SPAD, as shown in the setup schematic in Figure 2b. By increasing the driving current for the commercial LED, the background counts could be controlled. The modulated background optical signal was generated using a commercial 450 nm LED (LERTDUW S2W, OSRAM, Munich, Germany) modulated with a transistor. This commercial LED had a modulation bandwidth of 15.9 MHz, and was placed within a few centimetres of the transmitting LED. Modulating this LED with a PRBS effectively simulates operation of the correlation link in an environment with conventional optical wireless communication links.

### 4.2. Real Time Link

To demonstrate a practical system, an FPGA based synchronisation system was implemented, involving data transmission in frames consisting of a 6 bit clock word and 32 data bits. The clock word, “001101”, allows both frame level and symbol level synchronisation of data streams. Details on choice of clock work and synchronisation methods can be found in the Supplementary Materials of Reference [21]. A block diagram of the experimental setup for real-time transmission is shown in Figure 6b. On the transmitter side, the FPGA was used to generate a data stream in frames, with the 6 bit clock word. In contrast to the offline setup in Figure 2b, this FPGA directly supplied the falling edge trigger for the LED board, without the need for extra logic circuitry. The receiver FPGA (Xilinx Spartan-3, XEM3010, Opal Kelly, Portland, OR, USA) was connected to a separate PC, and clock synchronisation removed the need for a trigger from the transmitter. However, due to limitations from the FPGA boards, the achievable data rates with the real-time setup are limited to 20 kb/s. It should also be noted that the data rates quoted here include transmission of the clock word. This 18.75% overhead reduces useful data transfer to 8.42 and 16.84 kb/s for 10 and 20 kb/s links, respectively.

### 4.3. NANOBED Satellite Simulator Experiments

The LED transmitter and SPAD receiver systems were independently powered by separate NANOBED systems, positioned approximately 4 m apart. A 450 nm micro-LED was used, focussed on the receiver plane using an 8 mm focal length lens (C240TME-A, Thorlabs, Newton, NJ, USA), giving a pixel image size at of approximately 4 cm. To increase received power on the 20 μm diameter SPAD, a 35 mm focal length collection lens (ACL4532U-A, Thorlabs, Newton, NJ, USA) was used.

The satellite simulator test bed is a FlatSat-configured CubeSat system, which includes an electrical power system, batteries, an on-board computer and communication systems. A software design tool offers mission design, simulation and analysis, including a link to the hardware for in-loop simulation and testing. A software defined radio link to NANOBED enables ground software validation and operational testing, over which command and control of the system components can be invoked. The NANOBED EPS provides a 5 V bus suitable for powering the transmitter and receiver FPGA boards directly. For the SPAD supply, the unregulated battery bus was used with a voltage regulator to fix the voltage to 6 V.

## Figures and Tables

**Table 1:** Typical power requirements of the communication system.

Component	Voltage (V)	Current (mA)	Power (W)
Transmitter LED & electronics	5	181	0.905
Receiver electronics	5	122	0.610
Receiver SPAD	6	595	3.570
